# Efficient Deep Learning Model Compression for Sensor-Based Vision Systems via Outlier-Aware Quantization

**DOI:** 10.3390/s25092918

**Published:** 2025-05-05

**Authors:** Joonhyuk Yoo, Guenwoo Ban

**Affiliations:** 1 College of Information and Communication Engineering, Daegu University, Gyeongsan 38453, Republic of Korea; 2 NPS Co., Ltd., Cheongju 28371, Republic of Korea; gwban@npstech.co.kr

**Keywords:** quantization, outlier awareness, model compression, image feature extraction, sensor-based computer vision

## Abstract

With the rapid growth of sensor technology and computer vision, efficient deep learning models are essential for real-time image feature extraction in resource-constrained environments. However, most existing quantized deep neural networks (DNNs) are highly sensitive to outliers, leading to severe performance degradation in low-precision settings. Our study reveals that outliers extending beyond the nominal weight distribution significantly increase the dynamic range, thereby reducing quantization resolution and affecting sensor-based image analysis tasks. To address this, we propose an outlier-aware quantization (OAQ) method that effectively reshapes weight distributions to enhance quantization accuracy. By analyzing previous outlier-handling techniques using structural similarity (SSIM) measurement results, we demonstrated that OAQ significantly reduced the negative impact of outliers while maintaining computational efficiency. Notably, OAQ was orthogonal to existing quantization schemes, making it compatible with various quantization methods without additional computational overhead. Experimental results on multiple CNN architectures and quantization approaches showed that OAQ effectively mitigated quantization errors. In post-training quantization (PTQ), our 4-bit OAQ ResNet20 model achieved improved accuracy compared with full-precision counterparts, while in quantization-aware training (QAT), OAQ enhanced 2-bit quantization performance by 43.55% over baseline methods. These results confirmed the potential of OAQ for optimizing deep learning models in sensor-based vision applications.

## 1. Introduction

The rapid development of sensor technology and computer vision has significantly enhanced applications in autonomous driving, medical imaging, smart cities, and industrial automation. However, deploying deep neural networks on resource-constrained edge devices, IoT systems, and embedded sensor platforms remains a significant challenge due to the high memory and computational costs [1,2,3]. To address this, various model compression techniques have been proposed [4,5,6,7], with quantization emerging as a key method for reducing the computational complexity, model size, and power consumption [8,9,10,11,12]. By converting 32-bit floating-point weights into lower-precision representations, quantization enables real-time processing of sensor-based image data, making deep learning models feasible for edge-computing applications [13,14,15,16].

In embedded systems such as mobile devices, smart sensors, and autonomous edge platforms, low-precision quantization plays a critical role in reducing memory usage, computation latency, and energy consumption. However, applying quantization under limited precision below 8 bits (especially at 4 bits or lower) introduces significant challenges, including a loss of representation capacity, increased quantization error due to large dynamic ranges, and vulnerability to outliers that dominate weight distributions. These limitations often result in severe accuracy degradation when the quantized models are deployed on real-world embedded AI vision systems. Therefore, designing a low-precision quantization method that is both efficient and robust in handling outliers is essential for practical deployment.

One of the core challenges in low-precision quantization is maintaining model accuracy without retraining overhead. In particular, the post-training quantization (PTQ) methods often suffer from severe accuracy degradation at 4-bit precision or lower as they lack retraining to compensate for quantization errors. This issue is exacerbated by outliers in the weight distribution, which extend the dynamic range and decrease quantization resolution. In sensor-based vision tasks such as image classification, object detection, defect inspection, and 3D reconstruction, preserving fine-grained image features is crucial. However, outliers introduce quantization errors that significantly impact the quality of extracted features, degrading the overall performance of vision models.

This paper presents a novel outlier-aware quantization (OAQ) method, specifically designed to enhance sensor-based image analysis by mitigating the impact of outliers. OAQ effectively reshapes weight distributions using scaled weight normalization, narrowing the dynamic range and improving quantization resolution. Conventional quantization schemes [17,18,19,20,21,22], particularly in post-training settings, typically rely on uniform quantization grids determined by the minimum and maximum weight values within each layer or channel. These methods apply linear scaling and rounding operations to map full-precision weights into a limited set of discrete levels, often without accounting for the actual distribution of the weights. As a result, they suffer from performance degradation, especially in low-bit scenarios, due to the coarse quantization steps and the influence of distributional outliers. In contrast, our proposed OAQ method was designed to minimize this issue without introducing additional computational complexity or requiring retraining. OAQ can also be seamlessly integrated into both uniform and non-uniform quantization methods.

Recent studies have proposed distribution-aware quantization techniques to improve the performance of deep neural networks in low-precision settings. For example, DAQ [23] and DGQ [24] introduce channel- or group-wise strategies for reshaping weight distributions, while EdgeQAT [25] guides training using entropy-based signals for edge deployment. However, many of these methods require retraining or access to labeled data, making them less suitable for rapid deployment in data-constrained environments.

In contrast, the proposed OAQ method introduces a lightweight post-training framework that directly scales outliers using a simple, learnable factor, effectively reducing the quantization error while preserving the original distribution shape. Furthermore, our method incorporates perceptual similarity metrics (e.g., SSIM) to evaluate and reshape weight distributions more precisely, and it can be easily integrated into existing quantization pipelines (whether uniform or non-uniform) without retraining. This makes OAQ particularly suitable for efficient deployment on embedded sensor platforms. The experimental results demonstrated that OAQ significantly improved quantization performance across various CNN architectures, particularly in low-precision PTQ scenarios relevant to edge-based sensor applications.

The main contributions of this paper are as follows:

1. We introduce a novel perspective that perceptually approximating the trained weight distribution is critical for minimizing quantization error in post-training settings. To the best of our knowledge, this is the first work to apply the structural similarity (SSIM) index to weight distribution evaluation in quantization.
2. We propose outlier-aware quantization (OAQ), a lightweight post-training method that adaptively rescales weight outliers to reduce dynamic range distortion and improve quantization resolution, without requiring retraining or data access.
3. The proposed OAQ method is model-agnostic and compatible with a wide range of quantization schemes, including both uniform and non-uniform strategies. It can be seamlessly integrated into existing pipelines with negligible computational overhead.
4. Extensive experiments on multiple architectures and bit widths demonstrated that OAQ significantly improved performance, particularly under 4-bit quantization, compared with prior PTQ baselines.

The remainder of this paper is organized as follows. Section 2 reviews related works on quantization and sensor-based vision applications. Section 3 presents the proposed OAQ technique and its mathematical formulation. Section 4 details the experimental setup and performance evaluation. Finally, Section 5 concludes the paper.

## 2. Related Work

### 2.1. Outlier-Handling Techniques

Deep neural networks (DNNs) are highly sensitive to outliers in weight distribution, especially in low-precision quantization scenarios. Since the quantization process normalizes weights based on the maximum absolute value, outliers significantly expand the dynamic range, reducing the quantization resolution and degrading image feature extraction accuracy in sensor-based vision systems.

To mitigate this issue, Cai et al. proposed a weight-normalization-based quantization method, which reduces the range of the weight distribution by decreasing the maximum absolute value during training [26]. Similarly, Jung et al. proposed a quantization interval learning (QIL) technique, which learns the optimal outlier clipping threshold based on the model’s loss value, significantly improving the quantization performance [27]. However, these methods require training overhead, making them impractical for real-time sensor applications in embedded systems. Additionally, clipping-based methods suffer from clipping errors, where weights exceeding the threshold are mapped to zero, leading to loss of critical feature information in vision models.

To eliminate the need for retraining, Zhao et al. proposed an outlier channel splitting (OCS) quantization, which duplicates channels containing outlier weights at inference time, reducing the range of the weight distribution [28]. OCS suffers from severe performance degradation in low-bit quantization (≤4-bit), which is critical for embedded sensor applications. In another approach, Nagel et al. introduced data free quantization (DFQ), which leverages scale equivariance to minimize inter-channel weight distribution differences [29]. DFQ’s concept of scaling is somewhat related to our outlier-aware quantization (OAQ) technique in that both methods use a scaling approach. However, whereas DFQ applies scaling uniformly across all weights, OAQ specifically targets outliers, thereby reducing their negative impact while preserving the feature extraction accuracy of sensor-based vision models.

### 2.2. Uniform/Non-Uniform Quantization

Quantization techniques can be categorized as uniform and non-uniform according to the distance between quantization points [19,30]. In uniform quantization, all quantized values are evenly spaced, making it computationally efficient and widely used in hardware implementations [20]. Since trained weights generally follow a Gaussian-like distribution, uniform quantization is commonly applied [21].

However, non-uniform quantization can better accommodate the actual weight distribution, improving quantization efficiency. PoT (power-of-two)-based quantization generates denser quantization values near zero, leveraging the non-linearity of power functions [31]. In contrast, IPoT (inverse power-of-two)-based quantization applies an inverse square function to concentrate quantization values farther from the mean, improving energy efficiency by increasing weight sparsity [16,32,33,34]. Since both PoT and IPoT quantization methods rely on bit-shifting operations, they are highly hardware-friendly. However, optimally selecting non-uniform quantization values remains challenging as it requires prior knowledge of the statistical properties of the weight distribution, which may not be feasible in real-time sensor-based vision applications.

### 2.3. Quantization-Aware Training and Post-Training Quantization

Low-precision quantization (≤4-bit) often leads to severe accuracy degradation in sensor-based image analysis tasks as it reduces the ability of DNNs to extract meaningful features. Quantization-aware training (QAT) methods have been proposed to mitigate this issue by jointly optimizing quantization errors and inference losses [35,36,37]. QAT updates both the full-precision weights and quantization parameters during training, improving robustness to quantization errors. However, QAT has several limitations: (1) QAT requires an extensive retraining process, making it infeasible for real-time embedded sensor applications. (2) Many datasets required for QAT are proprietary or subject to General Data Protection Regulation (GDPR) restrictions [38]. Applying QAT to existing models requires access to original training data, which may not always be available.

To overcome these limitations, post-training quantization (PTQ) has been introduced [22]. PTQ enables immediate quantization without retraining, making it highly attractive for real-time sensor applications. However, low-bit PTQ (≤4-bit) suffers from severe accuracy degradation as it assumes a simplified weight distribution that does not always match the real characteristics of the trained model.

Recent advances have explored distribution-aware and adaptive quantization techniques to address accuracy degradation in low-bit neural networks. For instance, Hong et al. [23] proposed channel-wise distribution-aware quantization for super-resolution tasks, while Ryu et al. [24] introduced a group-wise distribution-aware quantization method tailored for diffusion models. Shen et al. [25] presented EdgeQAT, which incorporates entropy and distribution metrics to guide quantization-aware training for efficient edge deployment of lightweight LLMs. Although these methods target different problem domains, their emphasis on distribution-aware quantization aligns conceptually with our work. Unlike QAT-based methods that require retraining [23,24,25], our approach works in post-training settings and is particularly suitable for data-limited environments. Yang et al. [39] addressed data-free quantization by leveraging tiered collaborative learning. In addition, techniques such as adaptive bit width control [40] and sensitivity-aware mixed-precision quantization [41] have been shown effective in balancing model size and performance.

Compared with these approaches, this paper proposes a simple yet effective weight normalization-based quantization method, outlier-aware quantization (OAQ). Our proposed OAQ offers a lightweight, post-training method that specifically mitigates the negative impact of sparse outliers on quantization resolution, without requiring retraining or data access. Unlike existing PTQ methods, OAQ dynamically adjusts weight normalization to specifically target outliers, thereby enhancing quantization resolution and preserving image feature extraction accuracy in sensor-based applications. Our experimental evaluation demonstrated that OAQ effectively improved quantization performance across multiple CNN architectures, particularly in low-bit (3-bit, 4-bit) scenarios, where traditional PTQ methods struggled.

## 3. Outlier-Aware Quantization

This section first highlights the necessity of preserving the trained weight distribution in quantized models by analyzing the limitations of existing outlier-handling methods, particularly in post-training quantization (PTQ). Based on structural similarity (SSIM) index analysis, we then introduce outlier-aware quantization (OAQ), a novel method that effectively mitigates outliers using a scaling factor, thereby improving quantization resolution and enhancing sensor-based deep learning applications.

### 3.1. Motivation

**Figure 1:**
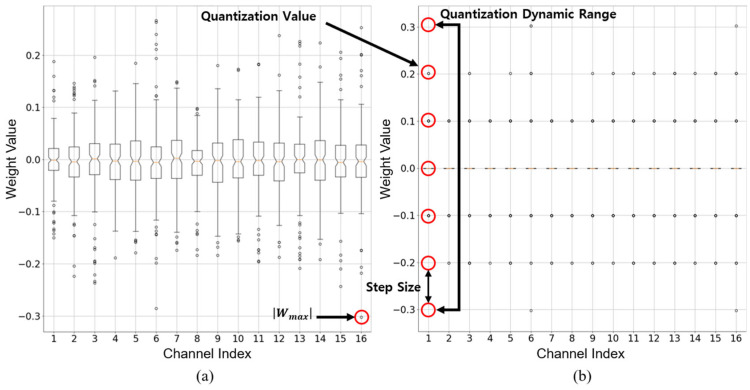
Performance degradation problem of vanilla quantization in ResNet20 [42]. (**a**) Channel-wise weight distribution of a 32-bit full precision layer. (**b**) Results of vanilla 3-bit uniform quantization applied to the same layer: due to a widened dynamic range caused by outliers, only a few of the seven available quantization values are effectively utilized.

In sensor-based vision models, accurate quantization of weights is crucial for preserving image feature extraction capabilities. However, trained weights exhibit layer- and channel-wise variations, leading to inconsistent quantization densities. Figure 1a illustrates the original channel-wise weight distributions in a specific layer of the trained 32-bit full-precision ResNet20 [42] model. These distributions serve as the reference for analyzing the impact of quantization. Figure 1b shows the outcome of applying vanilla 3-bit uniform quantization to the same layer. Due to the presence of extreme outliers in certain channels, the dynamic range expands, and many quantization levels are left unused in other channels, resulting in poor resolution and increased quantization error.

A primary cause of this issue is the presence of outliers, which are weights significantly larger or smaller than the mean of the distribution. Although outliers are rare, their influence is substantial, as illustrated in Figure 1. Uniform quantization assigns equally spaced quantization points centered at zero. However, in a 3-bit quantization scenario, as shown in Figure 1a, outliers force a wider dynamic range, thereby increasing the step size and reducing the quantization resolution. The total number of quantized values $n_{c}$ is defined as $n_{c}=2^{b}-1$. In this case, b=3, yielding $n_{c}=7$. However, due to the wide quantization intervals, most quantization values are not efficiently utilized, limiting the representation capability of the quantized DNN, as shown in Figure 1b.

To address the key challenges of low-bit quantization, such as reduced precision, quantization errors due to distribution outliers, and representational inefficiency caused by dynamic range expansion, we propose a scaled weight normalization-based outlier-aware quantization (OAQ) method that resizes weight distributions to mitigate the influence of extreme weights on resolution degradation. The proposed method applies a scaling factor to standard weight normalization, reducing the negative effects of outliers, narrowing the dynamic range, and ultimately increasing the quantization resolution.

**Figure 2:**
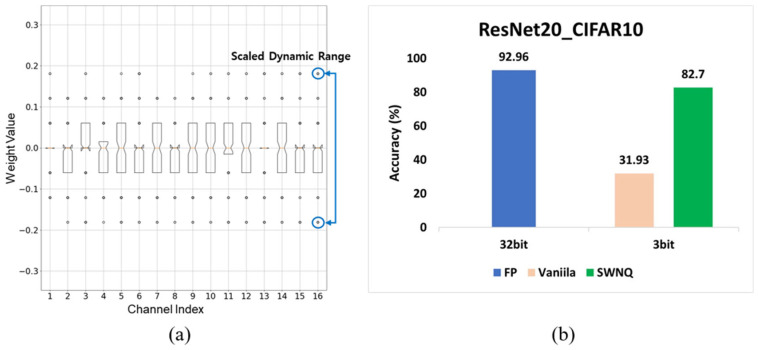
Effectiveness of OAQ in ResNet20 [42] quantization. (**a**) The 3-bit quantization result using OAQ: outlier scaling ensures that weights are evenly mapped across most quantization values, leading to higher resolution as the quantization step size narrows. (**b**) Accuracy comparison between the full-precision model, vanilla quantization, and OAQ: the outlier scaling mechanism in OAQ significantly enhances the low-precision quantization performance by improving the quantization resolution.

As demonstrated in Figure 2a, OAQ results in narrower dynamic ranges and step sizes compared with standard quantization. Consequently, OAQ ensures higher quantization resolution, allowing more efficient utilization of quantization levels. Additionally, as shown in Figure 2b, OAQ improves the 3-bit quantization accuracy by at least 60% over the baseline method, demonstrating its effectiveness in low-bit post-training quantization for edge-based vision models.

### 3.2. PTQ-Friendly Weight Distribution Reshaping

Convolutional neural networks (CNNs) play a fundamental role in sensor-based vision tasks, extracting and processing image features via convolutional layers. However, post-training quantization (PTQ) often degrades performance when converting full-precision models to low-bit models due to outlier-induced expansion of the dynamic range.

Several prior studies attempted to address this issue: ACIQ (analytical clipping for integer quantization) [21] identified the optimal clipping threshold based on bell-shaped weight distributions (Laplacian/Gaussian), minimizing the quantization error. However, since real-world weight distributions are not perfectly normal, ACIQ performs well at 8-bit precision but suffers severe degradation below 4-bit precision. DFQ (data-free quantization) [29] observed that cross-channel distribution variations cause quantization performance loss and proposed a cross-layer equalization method. However, DFQ modifies all weight scales equally, disregarding layer-specific outliers. GDRQ (generalized distribution reshaping quantization) [43] argued that previous quantization methods fail to reconstruct weight distributions properly and proposed a method for uniformly reshaping weight distributions. However, this approach still suffers from performance loss when applied in isolation and requires additional processing such as bias correction and per-group clipping threshold settings.

**Figure 3:**
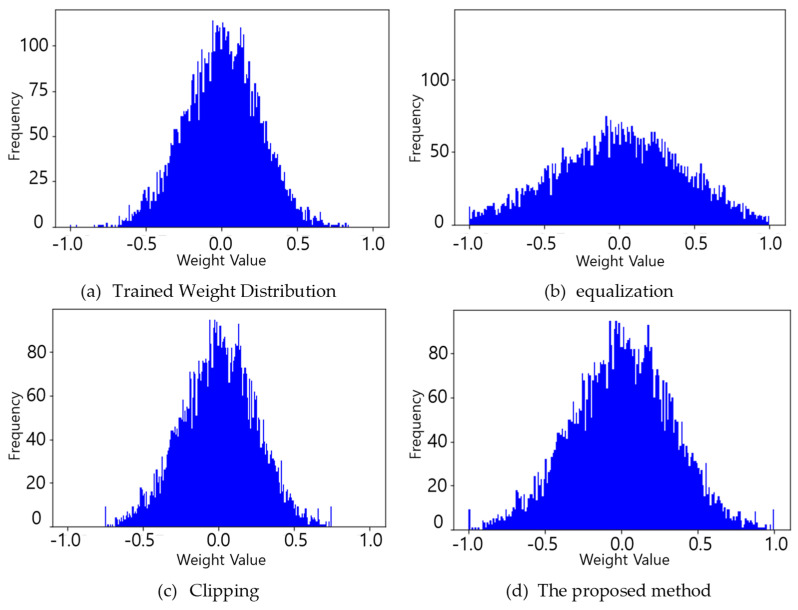
Per-layer quantization performance in ResNet20 [42] with and without outlier handling.

Figure 3 illustrates the effects of various outlier handling techniques on weight distributions. For the convenience of comparison, weight distributions are normalized to the range of [−1, 1]. Figure 3a is the trained weight distribution, and Figure 3b demonstrates per-channel scaling (DFQ [29]/GDRQ [43]), which alters the weight distribution shape. Figure 3c shows the results of clipping at ±0.75, preserving the in-threshold weight distribution but removing outlier information, potentially losing meaningful features. Last, Figure 3d presents the proposed OAQ-based weight scaling, which preserves the trained weight distribution while reducing outlier effects and results in maintaining important features.

Notably, in Figure 3d, the frequency at ±1 appears higher than in the original distribution (Figure 3a). This is because under the OAQ method, extreme weight values (i.e., outliers) are scaled and clipped to lie within the normalized range of [−1, 1]. As a result, values near the edges of the original distribution are mapped to ±1, contributing to the observed peaks. As shown in Figure 3c, applying hard clipping to the trained weight distribution may lead to abrupt changes in weight values, especially for outliers. We observed that this can cause a sharp drop in model performance during inference. Based on this observation, unlike hard clipping which completely discards outliers, the proposed OAQ retains their structure in a bounded form and preserves the overall shape of the weight distribution while reducing dynamic range distortion. Additionally, when comparing Figure 3a,d, it can be observed that the overall shape of the distribution is stretched under OAQ. This indicates that OAQ preserves the form of the trained weight distribution while enabling more weights to be mapped to a broader set of quantized values, thereby increasing the resolution and reducing the quantization loss.

To validate this approach, we quantitatively measured the outlier impact using the structural similarity (SSIM) index [13,14,15,44], a metric that evaluates distribution similarity between quantized and trained weights, which are defined by Equation (1).

(1) $$SSIM\left(x,y\right)=\frac{\left(2\mu_{x}\mu_{y}+c_{1})(2\sigma_{xy}+c_{2}\right)}{\left(\mu_{x}^{2}+\mu_{y}^{2}+c_{1})(\sigma_{x}^{2}+\sigma_{y}^{2}+c_{2}\right)}\;\;\;,$$

where x,y are the input image; $\mu_{x},$ $\mu_{y}$ are the mean of the two images; $\sigma_{x},\sigma_{y}$ are the standard deviation; and $\sigma_{xy}$ is the cross-covariance between the two images. The SSIM value ranges from 0 to 1; the closer it is to 1, the higher the similarity is.

To evaluate how well the quantized weights approximate the original trained weight distribution, we used the SSIM index as a perceptual metric. In our implementation, the weights were first normalized to the range [−1, 1] before the SSIM was computed. The calculation used a Gaussian window of size 11 × 11, consistent with conventional SSIM practices. The constants in the Equation (1) were set to $c_{1}={\left(0.01\times L\right)}^{2},\;c_{2}={\left(0.03\times L\right)}^{2}$, where L=2 is the dynamic range of the normalized weights. These parameter settings ensured consistent comparison across different quantization methods.

Although SSIM lacks invariance to geometric transformations such as scaling and rotation in image domains, this limitation did not affect its application in our context. Since we compared fixed, aligned 1D weight distributions without any reordering or transformation, the structural differences captured by SSIM remained meaningful and stable. Therefore, SSIM provided a perceptually relevant metric for quantifying the fidelity of weight structure after quantization.

Table 1 presents the SSIM analysis of different outlier handling techniques. The higher SSIM values between distributions indicate better distributional approximations of the trained model, while the lower SSIM values between weights indicate reduced redundancy between weights. As shown in Table 1, OAQ achieved a comparative higher distribution similarity (96.13%) while simultaneously reducing the redundancy of weights, which resulted in improved generalization performance of the quantized model.

In the proposed OAQ method, outliers were not removed but were rescaled and clipped to ±1 to prevent excessive expansion of the dynamic range. While this may result in multiple weights sharing boundary values, we observed through SSIM analysis that the overall shape of the distribution remained closer to the original than with hard clipping. Moreover, preserving these extreme values, albeit bounded, helped maintain the model’s representational diversity.

### 3.3. Handling Outliers with Scaling Factor

The objective of DNN quantization is to convert a set of full precision weights w∈W into a corresponding set of quantized weights $w^{Q}\in W^{Q}$, mapped to a predefined quantized value set $Q=\{q_{0},q_{1},...,q_{n}\}$. Depending to the quantization method, these values can be generated using uniform quantization or non-uniform quantization, such as the power-of-two (POT) and inverse power-of-two (IPoT) techniques [16,32,33]. While uniform quantization assigns evenly spaced intervals across the dynamic range, PoT and IPoT leverage non-linear quantization schemes to improve numerical efficiency and hardware compatibility.

(2) $$Q_{U}=w_{max}\times \left\{0,\pm \frac{1}{2^{b-1}-1},\pm \frac{2}{2^{b-1}-1},\ldots ,\pm 1\right\}\;Q_{PoT}=w_{max}\times \left\{0,\pm \frac{1}{2^{2^{b-1}-1}},\pm \frac{1}{2^{2^{b-1}-2}},...,\frac{1}{2},\pm 1\right\}\;Q_{IPoT}=w_{max}\times \left\{0,\pm \left(1-\frac{1}{2^{1}}\right),...,\pm \left(1-\frac{1}{2^{2^{b-1}-1}}\right),\pm 1\right\},$$

The dynamic range of quantization is determined by $w_{max}$, the maximum value of set W. In uniform quantization, the standard quantization method maps each weight to its corresponding quantized value using the following mapping function:

(3) $$w_{U}={\left|w\right|}_{max}\times \frac{\Pi \left(\hat{w}\times (2^{b-1}-1)\right)}{2^{b-1}-1}$$

The function $\Pi \left(\cdot \right)$ represents a rounding function that rounds values to the nearest integer. The normalized weight $\hat{w}$ is defined as a value in the range [−1, 1], obtained by dividing the original weight by ${\left|w\right|}_{max}=\max(\left|w\right|)$, the maximum absolute value, as shown in Equation (4):

(4) $$\hat{w}=\frac{w}{{\left|w\right|}_{max}},\;\;\;\hat{w}\in \left[-1,1\right]$$

The output of the $\Pi \left(\cdot \right)$ function is an integer within the range $\left[0,2^{b-1}-1\right]$. This projected integer weight is then converted into a uniformly quantized weight $w_{U}$ within the range $\left[-w_{max},w_{max}\right]$, as defined in Equation (3). The normalized weight $\hat{w}$ is commonly utilized in both uniform and non-uniform quantization methods to map weights to the corresponding quantized values in $Q_{U},Q_{PoT}$, and $Q_{IPoT}$.

Each weight distribution varies across layers and channels. During the initial step of the weight normalization process, most weights are scaled to significantly low values if an outlier in a specific layer or channel is substantially larger than the mean of the weight distribution. In addition, the dynamic ranges of weight distributions are highly inconsistent across channels, often due to the presence of sparse outliers. This channel-wise variation leads to inefficient global quantization step allocation and reduced resolution in narrow-range channels.

In training-based quantization, the set of full-precision weights W is optimized using a loss function based on the quantized weights $W^{Q}$ as the quantization process is applied only during forward propagation. In quantization-aware training (QAT), any loss of information caused by clipping or weight normalization can be compensated for during training. However, such compensation is not possible in post-training quantization (PTQ), where quantization is applied directly to pre-trained weights without additional fine-tuning.

This paper introduces a modified weight normalization-based PTQ method, which leverages a scaling factor to effectively manage outliers in the weight distribution. The modified quantization process begins with normalizing the full-precision weight w∈W by dividing it with the scaled maximum absolute value $\gamma {\left|w\right|}_{max}$. The resulting normalized weight $\hat{w}$ is clipped to the range [−1, 1] to ensure that all quantized values fall within the target dynamic range. The clipped and scaled value is then linearly mapped to an integer index in the quantization grid using a rounding function and finally scaled back to the original dynamic range. This whole process is mathematically expressed in Equations (5)–(7). The key intuition is that by introducing a tunable scaling factor before normalization, we can effectively compress the dynamic range of the weights while retaining structural information, thereby enabling better utilization of quantization bins.

(5) $$\hat{w}=clip\left(\frac{w}{\gamma {\left|w\right|}_{max}},1\right),\;\;\;\;\;\hat{w}\in \left[-1,1\right],$$

(6) $$clip(x,\alpha )=\left\{\begin{array}{c} x\;\;\;\;\;\;\;\;\;\;\;\;\;\;\;\;\;\;\;\;\;\;\mathrm{if}\;\;\;\;x\leq \alpha \\ sign(x)\cdot \alpha \;\;\;\;\;\;\mathrm{if}\;\;\;\;x>\alpha \end{array}\right.,$$

(7) $$w_{U}=\gamma {\left|w\right|}_{max}\times \frac{\Pi \left(\hat{w}\times 2^{b-1}-1\right)}{2^{b-1}-1},$$

Equation (5) shows how the normalized weights are computed by dividing each weight by the scaled dynamic range $\gamma {\left|w\right|}_{max}$. Since extreme weights may fall outside the [−1, 1] range after scaling, Equation (6) applies a clipping function to bound them within this interval. This step ensures that all weights are mapped to valid quantization bins, avoiding overflow and enabling higher resolution near the center of the distribution. Equation (7) defines the modified mapping from normalized weights to quantized values using the scaling factor γ, which adjusts the effective range of the quantization grid. Intuitively, a smaller γ compresses the dynamic range, thereby allowing the quantization process to assign more bins within the denser regions of the weight distribution. This leads to lower quantization error in central regions while still preserving the presence of outliers via clipping rather than discarding them entirely.

The OAQ process operates in two stages: (1) during fine-tuning, the scaling factor γ is iteratively optimized per layer to reshape the weight distribution; (2) during inference, the fixed γ is used to normalize, clip, and quantize the weights using the mapping function, described in Equations (5)–(7). This process is repeated per layer during inference or fine-tuning. This flow is detailed in Algorithm 1. OAQ operates in a model-agnostic fashion and does not alter the structure of original CNN architecture, making the proposed method efficient and easy to deploy.
**Algorithm 1: A Modified Weight Normalization-based PTQ Method Exploiting OAQ****Input:** W – The full-precision trained weights**Output:** $W^{Q}$– The quantized weights**1:****Procedure** FINE-TUNING**2:**Initialize weights to W**3:****for** l=1→L **do****4:**
${\hat{w}}_{l}⟵clip\left(\frac{w}{\gamma {\left|w_{l}\right|}_{max}},1\right),{\hat{w}}_{l}\in \left[-1,1\right],w_{l}\in W_{l}$**5:**
$w_{l}^{Q}⟵\gamma {\left|w_{l}\right|}_{max}\times \frac{\Pi \left({\hat{w}}_{l}\times 2^{b-1}-1\right)}{2^{b-1}-1},\gamma \in \left[0,1\right]$**6:**Compute the loss l**7:**Compute the gradient w.r.t. the output $\partial l/\partial x_{L+1}$**8:****for** l=1→L **do****9:**
Given $\partial l/\partial x_{l+1}$**10:**
Compute the gradient of the γ**11:**
Update the γ**12:**
Compute $\partial l/\partial x_{l}$**13:****Procedure** INFERENCE**14:**Initialize weights to W**15:****for** l=1→L **do****16:**
${\hat{w}}_{l}⟵clip\left(\frac{w}{\gamma {\left|w_{l}\right|}_{max}},1\right),{\hat{w}}_{l}\in \left[-1,1\right],w_{l}\in W_{l}$**17:**
$w_{l}^{Q}⟵\gamma {\left|w_{l}\right|}_{max}\times \frac{\Pi \left({\hat{w}}_{l}\times 2^{b-1}-1\right)}{2^{b-1}-1},\gamma \in \left[0,1\right]$**18:**
Deploy the quantized weights $W^{Q}$**19:****End**

Algorithm 1 outlines the modified weight normalization-based PTQ method following the above outlier-aware quantization (OAQ) consisting of normalization, clipping, and mapping processes. Lines 1–12 illustrate the fine-tuning process of the scaling factor γ in OAQ, and lines 13–18 describe the inference process of the standard PTQ method. The proposed OAQ is a simple yet effective approach that minimizes differences in the dynamic range of weights across channels while enhancing quantization resolution by applying the scaling factor to ${\left|w\right|}_{max}$.

### 3.4. Fine-Tuning of Scaling Factor

Addressing outliers using the scaling factor γ within the OAQ scheme can enhance the overall performance of both uniform and non-uniform quantization methods. The scaling factor γ can be set as a fixed hyper-parameter within the range [0, 1], depending on the bit precision of the quantization process. However, to further enhance its effectiveness, this paper fine-tunes γ by optimizing it as a trainable parameter for each layer in the DNN. This approach is necessary because the dynamic range of each layer is inherently determined by the layer-specific ${\left|w\right|}_{max}$.

To achieve this optimization, fine-tuning the scaling factor γ is performed using the straight-through estimator (STE) [34,45,46] within the back-propagation process, following the standard parameter learning techniques used in quantization. Although STE-based training can be unstable when applied to discrete-valued parameters, in our case, the STE is used solely to fine-tune the continuous scaling factor per layer. Since γ is a bounded scalar and not involved in discontinuous mappings, the risk of instability is significantly lower. In practice, we used a small learning rate and conservative schedule to ensure smooth convergence and did not observe any oscillations or divergence in training. It is worth noting that STE is a widely adopted technique in quantization-aware training and has become a de facto standard for handling non-differentiable operations during backpropagation. In our case, we applied STE only to optimize the scaling factor γ, bypassing the non-differentiability of rounding, which further reduced the instability risks compared with full QAT on weights and activations.

### 3.5. Measuring Quantization Sensitivity

The quantization sensitivity between a full-precision model and its quantized counterpart is typically evaluated by computing the sample mean of the Kullback–Leibler divergence (KLD), as defined by the following equation:

(8) $$\Omega \left(b\right)=\sum_{l=1}^{L}\Omega_{l}\left(b\right)=\sum_{l=1}^{L}KLD\left(M\left(w_{l};X\right),\;M\left(w_{l}^{Q}\left(b\right);X\right)\right)\;\;,$$

(9) $$KLD(P|\left|Q\right)=\sum_{j=1}^{N}P\left(j\right)\ln\left(\frac{P(j)}{Q(j)}\right)\;\;,$$

In Equation (8), Ω(b) represents the quantization sensitivity of the model, while $\Omega_{l}(b)$ denotes the quantization sensitivity of the *l*th layer quantized with b bits. The variable L refers to the total number of layers, $and\;W_{i}^{Q}(k)$ represents the weight of the *i*th layer quantized with k bits. In Equation (9), the Kullback–Leibler divergence (KLD) measures the difference in Shannon entropy between two probability distributions. This equation suggests that if a layer exhibits a lower $\Omega_{i}(k)$ value compared with other layers, it can be quantized with fewer bits since lower-precision quantization has minimal impact on overall performance. Conversely, if a layer has a higher $\Omega_{i}(k)$ value, the quantization loss becomes more siginificant, requiring a higher bit precision to performance degradation. To address this, the OAQ method adopts a mixed precision quantization approach, where the bit allocation per layer is determined based on quantization sensitivity measurements. This approach is implemented and evaluated in this paper.

## 4. Experiments

The proposed outlier-aware quantization (OAQ) method is implemented in the PyTorch (PyTorch ver.1.10.2) deep learning framework and evaluated by using 10,000 test images from the CIFAR-10 dataset. In this study, we focused on CIFAR-10 due to its widespread use in evaluating quantization and compression methods in low-resource scenarios. We also evaluated the proposed OAQ method on three widely used convolutional neural network architectures (ResNet20 [42], ResNet56 [42], and DenseNet100 [47]) to evaluate the robustness of OAQ across various model types. ResNet20 and ResNet56 are part of the ResNet [42] family, which employs residual connections to ease the training of deeper networks by mitigating vanishing gradients. ResNet20 has 20 layers and is a lightweight model often used for CIFAR-10, while ResNet56 is a deeper variant that offers more complex feature extraction. DenseNet100 is a densely connected convolutional network consisting of 100 layers, where each layer is connected to every other layer in a feedforward fashion. This architecture improves the parameter efficiency and feature reuse, making it robust to information loss during quantization. These models provide a representative benchmark for evaluating the quantization performance across varying levels of model depth and connectivity patterns.

The experimental setup consisted of a computing environment running on a server equipped with an Intel i7-6850K CPU, 64 GB DRAM, and an NVIDIA TITAN XP (12 GB GPU)(Manufactured by Asus Korea Co., Ltd., Seoul, Repulic of Korea). Training the 32-bit full-precision ResNet model, which was later subjected to post-training quantization (PTQ), took approximately 2 h for 200 epochs. The training time remained the same for quantization-aware training (QAT), whereas PTQ required no additional training since it directly quantized the pre-trained model.

To evaluate the effectiveness of the proposed outlier-aware quantization (OAQ), we applied our method exclusively to the vanilla quantization process, ensuring no additional modifications or enhancements. For clarity, we refer to the baseline comparison method as weight normalization-based quantization (WNQ) [48], distinguishing it as the standard vanilla quantization method to avoid terminology confusion.

### 4.1. Fixed Scaling Factor in Uniform and Non-Uniform OAQ

In this subsection, the scaling factor γ is treated as a fixed value, selected solely to assess the impact of outlier handling on performance improvement. The value of γ is adjusted in increments of ±0.01, starting from 0.5. Therefore, the scaling factor used here is not optimized. The fined-tuned, optimized scaling factor γ will be evaluated in the next subsection.

**Figure 4:**
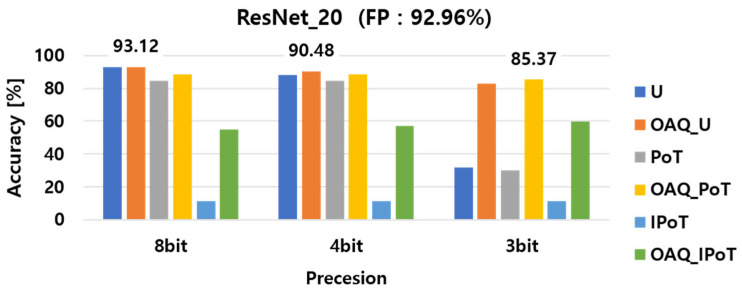
Per-layer quantization performance in ResNet20 with and without outlier handling.

**Figure 5:**
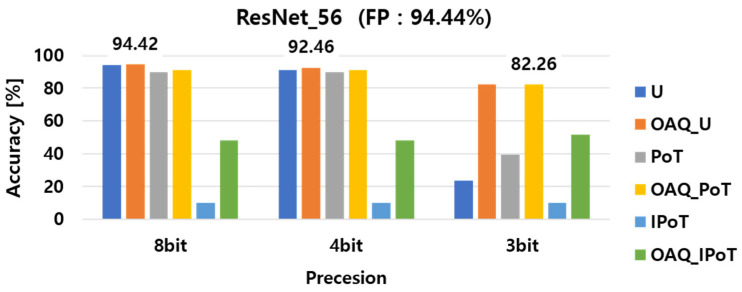
Per-layer quantization performance in ResNet56 with and without outlier handling.

**Figure 6:**
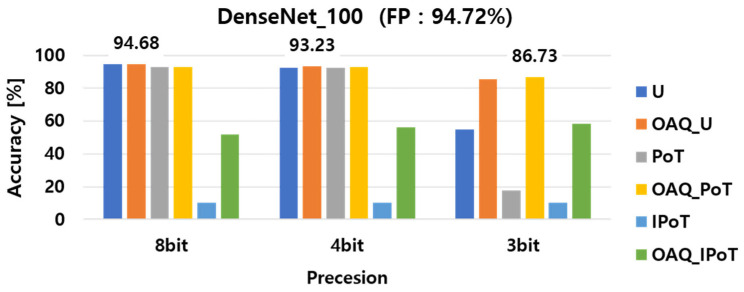
Per-layer quantization performance in DenseNet100 with and without outlier handling.

The experimental results of the implemented outlier-aware quantization (OAQ) for per-layer quantization performance, both with and without outlier handling, are presented in Figure 4, Figure 5 and Figure 6. For clarity, we denote each baseline quantization method as follows: U refers to vanilla uniform quantization; OAQ_U refers to OAQ applied to uniform quantization; PoT refers to power-of-two quantization; OAQ_PoT indicates OAQ applied to PoT; and similarly, IPoT and OAQ_IPoT refer to inverse power-of-two quantization and its OAQ-enhanced variant, respectively. First, we compared the accuracy of six different quantization configurations: uniform, uniform OAQ, PoT, PoT OAQ, IPoT, and IPoT OAQ. These methods were evaluated across three bit precision levels (8-bit, 4-bit, and 3-bit) and applied to three widely used DNN architectures: ResNet20, ResNet56, and DenseNet100. In the bar charts, the numerical values displayed above each cluster of bars indicate the highest accuracy achieved for the corresponding bit precision level among the six quantization methods.

As shown in Figure 4, Figure 5 and Figure 6, ResNet56 suffered more from performance degradation at 3-bit precision compared with ResNet20 and DenseNet100. This was due to its relatively narrower channel width, which amplified the effect of the reduced quantization resolution. ResNet56, while deeper than ResNet20, had narrower channels than DenseNet100 and exhibited greater sensitivity to precision loss when quantized at lower bit widths, and it could not compensate for the increased quantization error introduced in earlier layers, leading to worse accuracy. This supported our earlier observation that channel width, rather than depth or total parameter count, was a key factor in quantization robustness.

To provide a more precise comparison, Table 2 presents the classification accuracy of ResNet20, ResNet56, and DenseNet100 under 8-bit, 4-bit, and 3-bit quantization settings using the vanilla and OAQ methods. As shown, OAQ consistently outperformed vanilla quantization across all models and bit widths, with especially significant improvements at lower bit precisions. The performance gains observed with OAQ were particularly notable in the 3-bit setting, where vanilla quantization suffered severe degradation, while OAQ maintained relatively stable accuracy. These results reinforced the effectiveness of OAQ in maintaining accuracy under aggressive quantization, particularly for deeper or wider networks.

For all 8-bit precision models, the performance remained comparable to that of the full-precision model, with minimal degradation due to the high representation capability at this bit level. Interestingly, 8-bit OAQ achieved a significant improvement in ResNet20 accuracy, outperforming the full-precision model by approximately 0.2%, despite the quantization process. This result suggested that applying appropriate quantization to a DNN model could enhance its generalization performance, similar to the effect of adding controlled noise during training.

In contrast to the 8-bit precision case, significant performance degradation was observed in lower precision quantization, particularly at 4-bit and 3-bit precision. In 4-bit models, the accuracy of vanilla PTQ methods decreased by at least 2~4%, while in 3-bit models, performance dropped dramatically by 40~61% compared with the full-precision model. These results highlight a critical limitation of vanilla PTQ methods, which suffer from severe accuracy loss at bit precisions below 4 bits due to the absence of a retraining process. This issue served as the primary motivation for introducing outlier-aware quantization (OAQ) in this paper, addressing the challenges of low-bit quantization without retraining.

As shown in Figure 6, the DenseNet100 model exhibited less performance degradation compared with ResNet20 and ResNet56 when quantized at lower bit precision, particularly in 3-bit precision. The ResNet56 model, which had a greater number of parameters than ResNet20, demonstrated better quantization performance. However, despite having a similar parameter count to ResNet56, DenseNet100 experienced less performance degradation than ResNet20.

This variation in performance degradation across different DNN architectures during low-bit quantization was attributed to quantization sensitivity differences. Specifically, DenseNet100 exhibited lower quantization sensitivity than the other two models. These findings indicated that quantization sensitivity was not solely dependent on the number of parameters but was more closely correlated with the width of channels per layer. This result provided an important insight: models with wider channels per layer tended to have lower quantization sensitivity, making them more robust to low-bit quantization. Therefore, it was crucial to design an effective layer-wise quantization strategy by considering both the quantization sensitivity of the model architecture and the distribution of trained weights.

**Figure 7:**
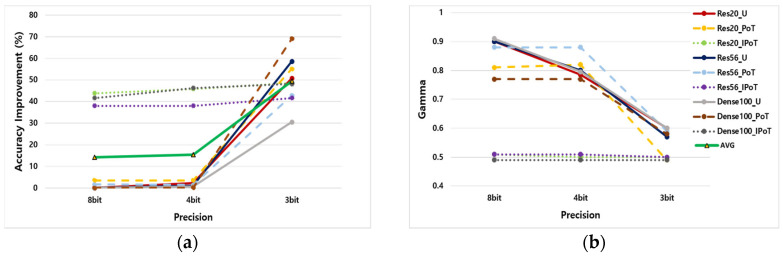
Contribution of the OAQ according to bit precision. (**a**) Magnitude of accuracy improvement by applying OAQ. The bold green line represents the average improvement across bit precisions. (**b**) Adjustment on scaling factor γ on bit precision.

Figure 7a illustrates the performance improvement achieved by the proposed OAQ in mitigating degradation across various quantized models. As the bit precision decreased from 8-bit to 3-bit, most quantized models exhibited enhanced accuracy, demonstrating the effectiveness of OAQ in addressing harmful outliers. The bold green line in Figure 7a represents the average performance improvement rate across different bit precisions. On 8-bit and 4-bit models, OAQ achieved an average accuracy improvement of 15%. However, the severe performance degradation of IPoT significantly influenced the overall results. Notably, at 3-bit precision, OAQ demonstrated a substantial advantage over vanilla PTQ methods, increasing the accuracy by an average of 49.5%.

Figure 7b illustrates the adjustable scaling factor γ in OAQ, which varied according to the bit precision. These results indicated that γ decreased as the bit precision decreased in both uniform and PoT quantization methods. This trend suggested that a narrower dynamic range was required to enhance quantization resolution as the number of available quantized values decreased at lower bit precision. Notably, in the PoT quantization method, the value of γ remained nearly the same at 8-bit and 4-bit precision. This was because PoT quantization inherently generated densely packed quantization steps near zero, making it more robust to outliers than uniform or IPoT quantization methods at 4-bit precision.

The vanilla IPoT quantization method exhibited exceptionally severe performance degradation, with accuracy dropping by more than 80% across all bit precisions. This issue arose from the inherent characteristics of IPoT, which sparsified most weights to zero while increasing quantization resolution at the outer regions of the weight distribution. Although this approach helped reduce energy consumption and enhance model compression, it posed challenges in training suitable weights in the PTQ setting.

To mitigate this issue, the proposed OAQ applied a significantly smaller scaling factor γ to the IPoT quantization compared with other methods. This adjustment shifted the outer weights closer to zero, ensuring that more weights were mapped to valid quantized values. By effectively gathering outliers around the upper and lower limits of a narrower dynamic range through clipping, OAQ significantly reduced the quantization error compared with the vanilla IPoT method.

**Figure 8:**
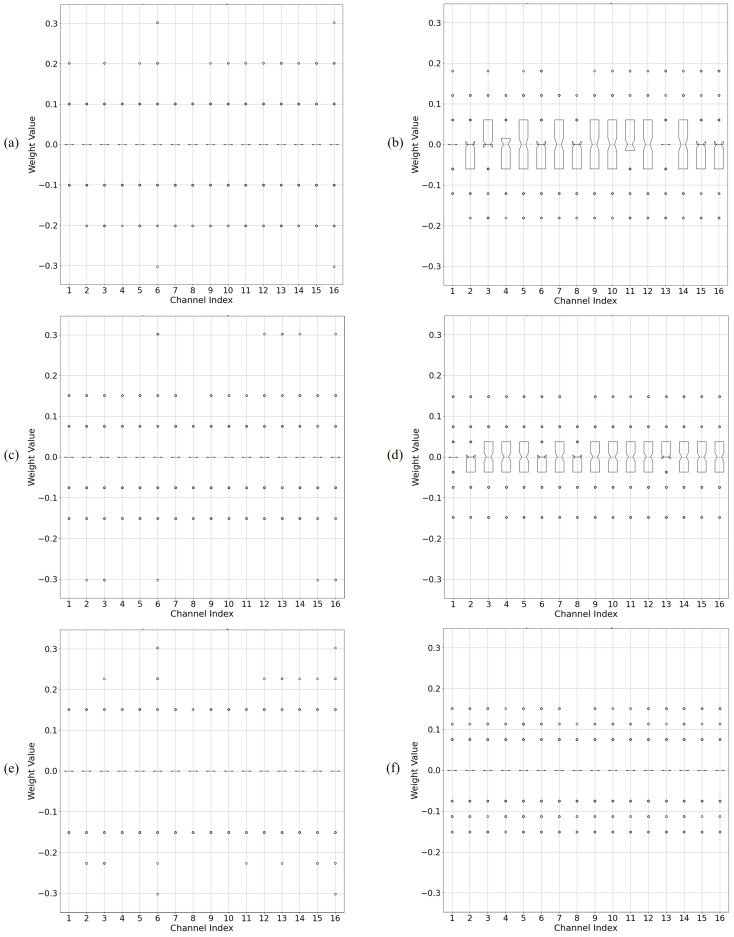
Enhancement of 3-bit quantization resolution in ResNet20 using OAQ by comparing 3-bit quantization resolution across three methods (uniform, PoT, and IPoT) with and without OAQ. The proposed OAQ achieved higher quantization resolution within a narrower dynamic range, ensuring more efficient utilization of quantization values. (**a**,**c**,**e**) Results of vanilla quantization for uniform, PoT, and IPoT, respectively. (**b**,**d**,**f**) Results of OAQ-enhanced quantization for uniform, PoT, and IPoT, respectively.

Figure 8 compares the quantized weight distribution of a ResNet20 layer between the vanilla PTQ method and the proposed OAQ. Per-layer quantization methods that assigned the same quantization values across layers demonstrated the significant impacts of outliers. In the vanilla PTQ method, a substantial portion of the available quantization values remained unused in certain channels with a narrow dynamic range, leading to inefficiencies. This issue arose because outliers in specific channels expanded the dynamic range, causing a disproportionate allocation of quantization levels.

In contrast, the proposed OAQ effectively managed outliers using a scaling factor, ensuring that the quantization dynamic range remained narrow. As a result, weights were evenly distributed across a broader range of quantization values, thereby enhancing quantization resolution. This improvement was particularly evident in the IPoT scheme, where OAQ optimally redistributed weights, maximizing the utilization of quantization levels.

These experimental results demonstrated that the proposed outlier-aware quantization (OAQ) was a simple yet highly effective approach for enhancing quantization resolution by addressing outliers. By adaptively scaling the dynamic range through adjustments to the scaling factor γ, OAQ effectively mitigated the performance degradation observed in previous post-training quantization (PTQ) methods, particularly in low-bit precision scenarios.

### 4.2. Fine-Tuning the Scaling Factor for Optimization

In the following subsection, the scaling factor γ is optimized through fine-tuning, different from the previous setting. To deeply analyze the inner mechanisms of OAQ, say the outlier impact, scaling dynamics, and quantization sensitivity, we used ResNet20 as a reference model afterward due to its compact architecture and interpretability.

Table 3 compares the performance of several state-of-the-art (SOTA) quantization methods. Notably, with the exception of ZeroQ [38], OCS [28], and the proposed OAQ, all other quantization methods were quantization-aware training (QAT)-based approaches, requiring a full retraining process for the weights. In contrast, OAQ trained only the scaling factor γ, eliminating the need for weight retraining. In Table 3, although OAQ showed slightly lower performance than the method proposed in [38], it is important to note that [38] employed a data-aware quantization strategy with a lightweight retraining phase, whereas our method operated in a fully data-free post-training setting. This trade-off highlights the practicality of OAQ in scenarios where training data are unavailable or retraining is infeasible. The key contribution of this work lies not in outperforming all methods in absolute accuracy but in achieving significant performance restoration within a PTQ pipeline under low-bit settings. The last column in Table 3 shows the relative performance change, say the degree of degradation (−) or improvement (+) before and after applying quantization.

In addition to prior methods, we also compared with more recent quantization techniques [24,25,39,40,41], including DGQ [24], EdgeQAT [25], and sensitivity-aware mixed precision approaches [41]. While many of these methods rely on additional retraining phase, OAQ maintains competitive accuracy in a pure post-training scenario with negligible overhead.

For fine-tuning γ, the following hyper-parameters were used: the batch size was set to 128, and the initial learning rate was set to 2e-5, which decreased by 10% every 30 epochs. The training duration was up to 100 epochs. Furthermore, since OAQ primarily focused on weight quantization, fine-tuning was applied only to weights, while activation quantization remained heuristic, as detailed in Section 4.1.

On ResNet20, all QAT-based methods required more than 2 h to train the quantized weights, whereas OAQ completed training in less than 20 min since only the scaling factor γ was fine-tuned. From a parameter efficiency perspective, the QAT-based methods involved training of 0.27M parameters, while OAQ only required learning one parameter per layer, significantly reducing the computational overhead. Fine-tuning had minimal impact at 8-bit precision as the high representation capability prevented significant performance degradation. However, OAQ demonstrated its effectiveness even at the 8-bit setting by achieving 0.52% better accuracy than the fully trained full-precision (FP) models, despite not undergoing fine-tuning.

The effectiveness of outlier-aware quantization (OAQ) in 4-bit precision experiments was particularly notable. The results clearly demonstrated the impact of outlier scaling as OAQ (w/o FT) produced fewer quantization errors than OCS [28] in PTQ settings without fine-tuning. Furthermore, OAQ outperformed OCS and even exceeded the performance of DoReFa-Net [19], a quantization-aware training (QAT)-based method, when fine-tuning was applied.

Notably, OAQ (w/FT) achieved a performance gap −0.64% in quantization error while surpassing APoT [33], a QAT-based method, by +0.36% under the same weight and activation bit precision settings. This result is a significant contribution of this paper, demonstrating that the post-training quantization (PTQ)-based OAQ achieved performance comparable to state-of-the-art (SOTA) QAT-based methods. These findings illustrated that the outlier scaling approach in OAQ was quantization-friendly and effectively enhanced PTQ performance by reconstructing a weight distribution that closely approximated the trained model.

### 4.3. OAQ with Mixed-Precision Quantization

**Figure 9:**
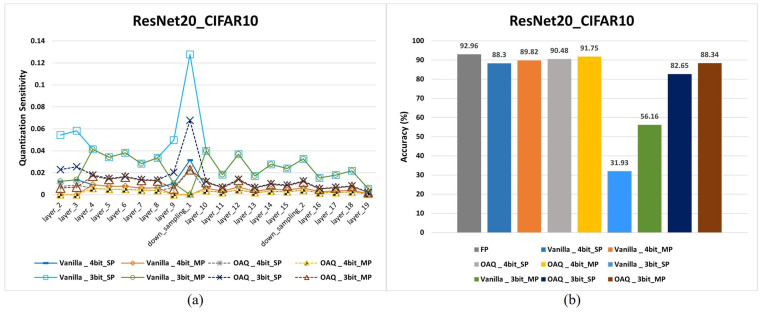
Comparison of vanilla quantization and OAQ for mixed-precision (MP) quantization. Applying OAQ alongside mixed-precision quantization reduces quantization sensitivity and improves performance. (**a**) Sensitivity comparison of quantized ResNet20 on CIFAR10: OAQ effectively reduces quantization sensitivity, leading to improved stability. (**b**) Accuracy comparison of quantized ResNet20 on CIFAR10: OAQ enhances accuracy, particularly in low-bit quantization scenarios. SP: single precision; MP: mixed precision.

This subsection analyzes mixed-precision OAQ, which leverages layer-wise quantization sensitivity to optimize quantization performance. Figure 9a illustrates the layer-wise quantization sensitivity of the quantized ResNet20 model on the CIFAR10 dataset. In Figure 9, single-precision (SP) refers to a setting where all layers are quantized with the same bit precision, whereas mixed-precision (MP) indicates a configuration where each layer is quantized with a different bit precision, adapting to its sensitivity characteristics.

When the quantization sensitivity was sorted in ascending order for both 3-bit and 4-bit precision, four layers (2, 3, 9, and down_sampling_1) exhibited higher sensitivities than other layers in the single-precision (SP) setting. This observation suggested that these layers should be quantized with higher bit precision to mitigate accuracy loss. To implement a mixed-precision (MP) approach, these four high-sensitivity layers were assigned 8-bit precision, while the remaining layers retained lower-bit quantization.

Figure 9a illustrates that the overall quantization sensitivity of the proposed OAQ was consistently lower than that of the vanilla method. This result confirmed that OAQ effectively reduced the quantization sensitivity by properly managing outliers through the scaling factor, thereby improving the quantization robustness. Figure 9b compares the performance of vanilla quantization and OAQ under uniform SP and MP quantization settings. In the 4-bit SP quantization scenario, the proposed OAQ achieved a performance improvement of approximately 2.2% compared with the vanilla method. Furthermore, when MP-based quantization was applied, assigning 8-bit precision to layers with high quantization sensitivity, OAQ achieved an accuracy of 91.75%, resulting in a performance gap of only 1.2% compared with the 32-bit full-precision model.

However, at 3-bit precision, performance degradation was severe, as commonly observed in post-training quantization (PTQ) methods. When SP quantization was applied, the proposed OAQ experienced significantly lower performance degradation, approximately 10%, compared with the 61% degradation of the vanilla method, relative to the 32-bit full-precision model. When MP quantization was introduced, OAQ further reduced the performance gap to just 4%, whereas the vanilla method still suffered from a substantial 37% degradation compared with the full-precision model. These results confirmed that the proposed OAQ was highly effective in low-precision, MP-based PTQ, significantly mitigating accuracy loss in extreme quantization scenarios.

### 4.4. Integrating OAQ with Quantization-Aware Training

The proposed OAQ can be integrated with the quantization-aware training (QAT) method to investigate whether the impact of outliers on quantization extends beyond post-training quantization (PTQ) to QAT as well. A key advantage of QAT is its ability to simultaneously optimize both weights and quantization parameters, enhancing the performance of the quantized model. However, in this experimental setting, we trained only the weights while keeping the scaling factor γ fixed, allowing us to specifically evaluate the effectiveness of outlier scaling in the quantization process.

Table 4 demonstrates that even in a quantization-aware training (QAT) setting, the proposed outlier-handling technique significantly enhanced the quantization performance. Notably, OAQ enabled stable quantization down to 2-bit precision, achieving a 43.55% improvement, with an accuracy reduction of only 2.46% compared with the full-precision (FP) model.

The QAT-based OAQ was implemented by training the ResNet20 model on the CIFAR10 dataset. The batch size was set to 128, and the weight decay was set to 2e-4. Each model was trained for up to 150 epochs, with the learning rate initially set to 3e-5 and progressively decayed by a factor of 10 at epochs 40, 80, and 120. This gradual learning rate reduction helped progressively bridge the gap between the full-precision model and the quantized model. To further stabilize training, we progressively trained models at different bit precisions using the following sequence: 32-bit → 4-bit → 3-bit → 2-bit. The scaling factor γ for each bit precision was determined based on Figure 7b and set as 0.85 at 4-bit, 0.7 at 3-bit, and 0.5 at 2-bit precision.

**Figure 10:**
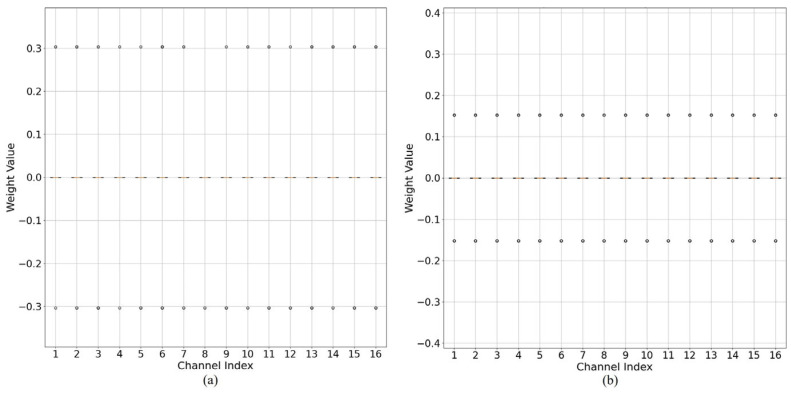
Comparison of weight distributions in the vanilla and OAQ methods for quantized models in QAT. (**a**) Weight distribution in the vanilla 2-bit quantization method, which shows a wider dynamic range with inefficient quantization. (**b**) Weight distribution in OAQ-based 2-bit quantization, which demonstrates a narrower dynamic range, ensuring more efficient weight mapping and improved quantization resolution.

Figure 10 illustrates the weight distribution in both the vanilla and OAQ-based methods. In OAQ, quantization values were mapped within a much narrower dynamic range compared with the vanilla method. This result confirmed that the outlier scaling technique effectively reduced the dynamic range, making the quantization process more stable and facilitating quantization-friendly weight training.

From a deployment perspective, OAQ introduced negligible overhead since the only added operation was a per-layer scaling factor applied prior to quantization. The quantized model size was reduced proportionally to the target bit width, say 4-bit quantization yields 8× memory savings, while OAQ preserved accuracy without requiring full QAT retraining. Thus, OAQ is suitable for edge devices and hardware accelerators where low-latency, low-memory inference is critical.

## 5. Conclusions

This paper introduces outlier-aware quantization (OAQ), a novel method to mitigate performance degradation in low-precision quantization by effectively handling outliers. Our analysis revealed that sparse outliers extended the dynamic range of weights, leading to reduced quantization resolution and accuracy loss. By leveraging the structural similarity (SSIM) index, we demonstrated that approximating the trained weight distribution was critical for improving post-training quantization (PTQ) at low-bit precision.

The proposed OAQ directly mitigated the influence of extreme weights on resolution degradation and applied a scaling factor that reduced the dynamic range while evenly redistributing weights, enhancing the quantization resolution without requiring additional computational complexity. Unlike previous weight scaling and clipping methods, OAQ effectively balanced outlier suppression and weight preservation, ensuring more stable and accurate low-bit quantization.

The experimental results validated the superiority of OAQ over existing PTQ methods, particularly in extreme low-bit settings (≤4-bit). Furthermore, OAQ can be seamlessly integrated into existing quantized DNN models without retraining or excessive computational cost, making it a practical and efficient solution for real-world deep learning applications. By demonstrating competitive performance even against state-of-the-art QAT-based methods, this study highlights the significance of outlier handling in quantization and paves the way for further research in high-efficiency model compression techniques for sensor-based on-device AI vision systems.

Despite its advantages, the proposed OAQ method has several limitations and practical considerations for deployment in real-world embedded systems. First, although the method introduced minimal computational overhead, storing a layer-wise or channel-wise scaling factor may incur memory cost in highly resource-constrained environments. Second, depending on the target hardware, additional scaling operations may affect inference latency, particularly in platforms with limited support for floating-point arithmetic. Finally, integrating OAQ with integer-only or hardware-specific quantization pipelines may require additional implementation optimization. Last, while our experiments on CIFAR-10 demonstrated the effectiveness of OAQ, further validation on large-scale and diverse datasets such as ImageNet and COCO and broader architecture families like lightweight or transformer-based models is necessary to confirm its generalizability for a more comprehensive assessment. These limitations should be carefully addressed in future work to facilitate efficient and scalable deployment of OAQ in real-world embedded applications.

While OAQ effectively improves the quantization robustness against internal distributional noise such as weight outliers, analyzing its behavior under external perturbations such as sensor noise or adversarial attacks remains an open direction. Future work will investigate OAQ’s applicability to adversarial robust quantization and its integration with noise-aware training techniques.

## Figures and Tables

**Table 1:** Structural similarity (SSIM) index comparison of different outlier handling methods.

Methods	SSIM Index Between Distributions [%] ↑	SSIM Index Between Weights [sum] ↓
Trained weight distribution	100	1.5942
Equalization (DFQ/GDRQ)	80.85	0.0765
Clipping (ACIQ)	99.99	1.5951
Proposed OAQ	96.13	0.9976

**Table 2:** Accuracy results (%) across models, quantization methods, and bit precisions. The vanilla method means uniform quantization, and the OAQ method means OAQ applied to uniform quantization.

Model	Method	8-bit	4-bit	3-bit
ResNet20	Vanilla	92.9	88.0	36.5
ResNet20	OAQ	93.1	90.5	85.4
ResNet56	Vanilla	94.1	90.6	23.7
ResNet56	OAQ	94.4	92.5	82.3
DenseNet100	Vanilla	94.6	92.3	55.3
DenseNet100	OAQ	94.7	93.2	86.7

**Table 3:** Accuracy comparison of existing quantization methods and the proposed OAQ.

Methods	Precision (Bit Widths of Weights/Activations)	Accuracy (%) (FP-32/Quantized)	Relative Performance Change Before and After Applying Quantization (%)
ZeroQ [38]	8-bit/8-bit	94.03/93.94	−0.09
OAQ (w/o FT)		92.60/93.12	+0.52
OAQ (w/FT)		**92.60/93.10**	**+0.55**
APoT [33]	4-bit/8-bit	91.60/92.30	+0.70
OAQ (w/o FT)		92.60/90.51	−2.09
OAQ (w/FT)		**92.60/92.66**	**+0.06**
OCS [28]	4-bit/4-bit	92.96/89.10	−3.86
DoReFa-Net [19]		91.60/90.50	−1.10
PACT [20]		91.60/91.70	+0.10
OAQ (w/o FT)		92.60/89.66	−2.94
OAQ (w/FT)		**92.60/92.61**	**+0.01**

FP: full precision; FT: fine-tuning.

**Table 4:** Accuracy comparison of vanilla quantization and OAQ in quantization-aware training (QAT) with 32-bit activations.

Methods	Precision (Weights)	Accuracy (%) [FP: 92.60%]
**Vanilla**	4-bit	92.64
	3-bit	90.91
	**2-bit**	**46.59**
**OAQ**	4-bit	92.79
	3-bit	92.12
	**2-bit**	**90.14**

## Data Availability

All datasets used in this article are publicly accessible.

## Abbreviations

The following abbreviations are used in this manuscript:

DNN	Deep neural network
CNN	Convolutional neural network
QAT	Structural similarity
OAQ	Outlier-aware quantization
PTQ	Post-training quantization
QAT	Quantization-aware training
QIL	Quantization interval learning
OCS	Outlier channel splitting
DFQ	Data free quantization
PoT	Power-of-two
IPoT	Inverse power-of-two
GDPR	General data protection regulation
ACIQ	Analytical clipping for integer quantization
STE	Straight-through estimator
KLD	Kullback–Leibler divergence
SP	Single precision
MP	Mixed precision
FP	Full precision
FT	Fine-tuning
